# Stable mammalian expression of His-tagged prestin in Chinese hamster ovary cells

**DOI:** 10.1007/s10616-026-00950-8

**Published:** 2026-04-10

**Authors:** Yasunori Donjo, Hisashi Sugimoto, Ryosei Motoo, Manabu Inaba, Tomokazu Yoshizaki, Michio Murakoshi

**Affiliations:** 1 Department of Otolaryngology-Head and Neck Surgery, Kanazawa University, Kanazawa, Japan; 2 Faculty of Frontier Engineering, Institute of Science and Engineering, Kanazawa University, Kanazawa, Japan

**Keywords:** CHO cells, Stable expression, Membrane protein production, His tag, Prestin

## Abstract

Chinese hamster ovary (CHO) cells are widely used for the stable production of recombinant proteins, including membrane proteins that require a mammalian cellular environment for proper folding and targeting. Prestin, a motor protein responsible for outer hair cell electromotility in the mammalian cochlea, is a multi-pass membrane protein whose structural and functional analyses require reliable expression systems capable of producing full-length protein. In the present study, we established CHO cell lines stably expressing full-length prestin with a C-terminal 6×histidine (His) tag using mammalian expression vectors driven by either the elongation factor-1α (EF1α) promoter or the cytomegalovirus (CMV) promoter. Following geneticin selection and limiting dilution cloning, multiple clonal cell lines were obtained and characterized by Western blotting, immunofluorescence microscopy and electrophysiological analysis. Seven clones expressing His-tagged prestin were identified. Quantitative Western blot analysis using a calibrated His-tagged reference protein demonstrated that the EF1α-driven system yielded higher-producing clones than the CMV-driven system, with a maximum estimated production of approximately 271 µg per 2 × 10⁹ cells. Immunofluorescence imaging confirmed membrane localization of prestin in expressing clones. Whole-cell patch-clamp recordings revealed nonlinear capacitance, indicating functional activity of the expressed protein. These results demonstrate that CHO cells provide a stable mammalian expression system for the production of full-length His-tagged prestin. The system enables reproducible protein production, quantitative expression evaluation and functional validation within a single cellular background, and may facilitate future biochemical, structural and functional studies of prestin and related membrane proteins.

**Supplementary Information:**

The online version contains supplementary material available at 10.1007/s10616-026-00950-8.

## Introduction

Outer hair cells (OHCs) in the mammalian cochlea exhibit electromotility, which plays a critical role in cochlear amplification and hearing sensitivity (Brownell et al. 1985; Ashmore 1987; Kachar et al. 1986; Dallos 1992; Liberman et al. 2002; Wu et al. 2004). This electromotility is driven by prestin, a membrane motor protein densely localized in the lateral membrane of OHCs (Dallos et al. 1991; Forge 1991; Huang and Santos-Sacchi 1993). Extensive efforts have been made to clarify its structural organization and functional mechanisms since the identification of prestin as the molecular basis of OHC electromotility.

Prestin is a multi-pass membrane protein belonging to the SLC26 anion transporter family (Zheng et al. 2000). Because of its complex membrane topology and oligomeric assembly, structural and biochemical analyses require reliable expression systems capable of producing full-length prestin in a membrane environment compatible with proper folding and trafficking. Various expression approaches have been explored, including bacterial expression of soluble domains, insect-cell systems and transient mammalian expression (Zheng et al. 2005, 2006; Navaratnam et al. 2005; Greeson et al. 2006; Detro-Dassen et al. 2008; Wang et al. 2010; Hallworth and Nichols 2012; Mio et al. 2008; Futamata et al. 2022; Butan et al. 2022; Ge et al. 2021; Bavi et al. 2021; Kumano et al. 2010; Murakoshi et al. 2006, 2009). However, these systems often fail to produce full-length prestin in a form that is correctly folded and targeted to the membrane, and they may show variability in expression levels between experiments or passages, making it difficult to achieve stable long-term protein production.

Stable mammalian expression systems offer several advantages for membrane protein production, including consistent expression levels, reproducible cell populations and compatibility with downstream biochemical and functional analyses. Chinese hamster ovary (CHO) cells are widely used for recombinant protein production due to their robustness in culture and suitability for stable clone selection.

We previously established CHO cell lines stably expressing prestin with FLAG (Iida et al. 2003) and Avi tags (Murakoshi and Wada 2021), demonstrating the feasibility of mammalian stable expression for this motor protein. While these tagging strategies are useful for detection and purification, they often require specialized reagents and may increase experimental cost. In contrast, the 6×histidine (His) tag is widely used for protein purification because of its small size and compatibility with immobilized metal affinity chromatography.

In the present study, therefore, we developed CHO cell lines stably expressing full-length prestin with a C-terminal His tag using expression vectors driven by either the elongation factor-1α (EF1α) promoter or the cytomegalovirus (CMV) promoter. Clonal cell lines were established by drug selection and limiting dilution, and prestin expression was evaluated by Western blotting and immunofluorescence microscopy. Functional activity of the expressed protein was also examined using whole-cell patch-clamp recordings. Together, these features establish a stable mammalian expression system for His-tagged prestin that enables reproducible production, quantitative evaluation and functional validation within a single cellular background, thereby facilitating future structural and electrophysiological studies of prestin.

## Materials and methods

### Gene construction

The pCR-Blunt II-TOPO cloning vector (Invitrogen, Carlsbad, CA) containing prestin cDNA cloned from the gerbil cochlea (Accession No. AF230376.2), which was developed in our previous study (Iida et al. 2003), was used as a template. Prestin cDNA was amplified from this template by polymerase chain reaction (PCR) using two sets of primers (Nos. 1 and 2, Table 1). Two types of prestin cDNAs with distinctive 15-base ends were developed. These terminal sequences were designed to be homologous to those of the linearized pBApo-EF1α Neo DNA and pBApo-CMV Neo DNA expression vectors (TaKaRa Bio, Shiga, Japan), enabling the subsequent In-Fusion cloning procedure. These vectors incorporate either a human elongation factor 1-alpha (EF1α) promoter or a cytomegalovirus (CMV) promoter.

Different sets of primers (Nos. 3 and 4, Table 1) were prepared for the EF1α and CMV expression vectors, respectively. These vectors were linearized by inverse PCR with a thermal cycler (T100 Thermal Cycler, Bio-Rad, Hercules, CA).

Amplified prestin cDNAs and linearized vectors were treated with the Cloning Enhancer (TaKaRa Bio), and prestin cDNAs were inserted into the multiple cloning site (MCS) of the EF1α and CMV expression vectors by In-Fusion (TaKaRa Bio).

The constructed expression vectors were subjected to mutagenesis PCR with a set of primers to add the His tag to the C terminus of prestin. The 15 base pairs of the 5’ ends of these primers overlapped with each other and contained the DNA sequence of 6×His within the overlapping region (No. 5, Table 1).

**Fig. 1:**
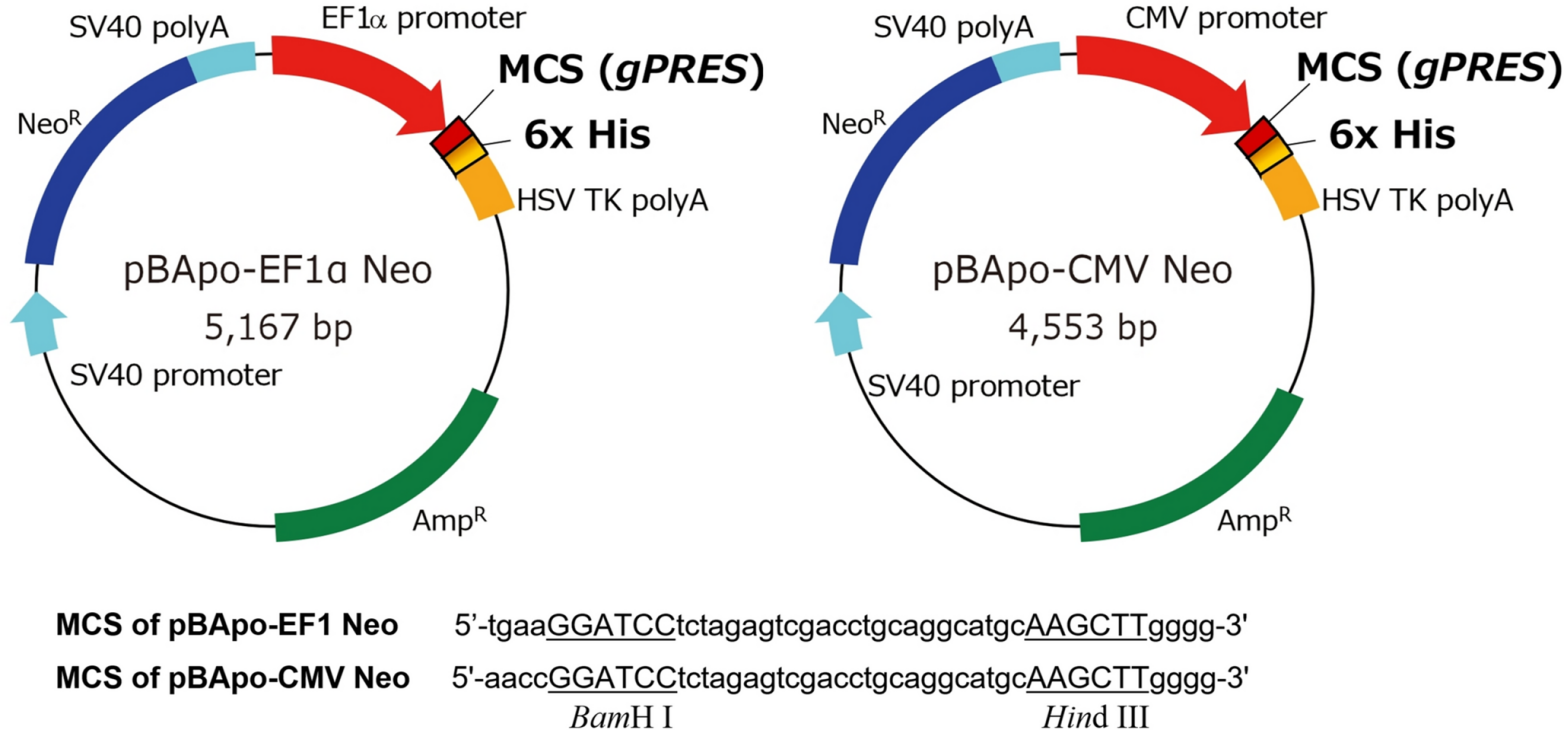
Design of mammalian expression vectors for the establishment of a stable cell line expressing His-tagged prestin. To achieve high expression levels, two types of vectors were constructed, each containing the EF1α or CMV promoter. In addition, both vectors carry a neomycin resistance gene to enable the selection of stable transfectants. The gene of gerbil prestin, fused with a 6×His tag at its C terminus, was inserted into the multiple cloning sites (MCS) of the expression vectors between the *Bam*H I and *Hin*d III restriction sites

The constructed expression vectors were transformed into *Escherichia coli*-competent cells. Transformed *E. coli* was cultured in a LB medium plate supplemented with ampicillin (50 µg/ml). After the incubation, the expression vectors were extracted from *E. coli* using the Gen Elute Plasmid Miniprep Kit (Sigma-Aldrich, St. Louis, MO). The extracted vectors were analyzed using an automated DNA sequencer with primers designed to check the down- and upstream regions of prestin (No. 6, Table 1). Figure 1 shows the design of the expression vectors used in this study.

The Kanazawa University Safety Management Regulation for Genetic Recombinant Experiments, Kanazawa, Japan, approved the experimental protocols for this study (number: Kindai 6–2237). All procedures were completed in accordance with the relevant guidelines and regulations.

**Table 1:**
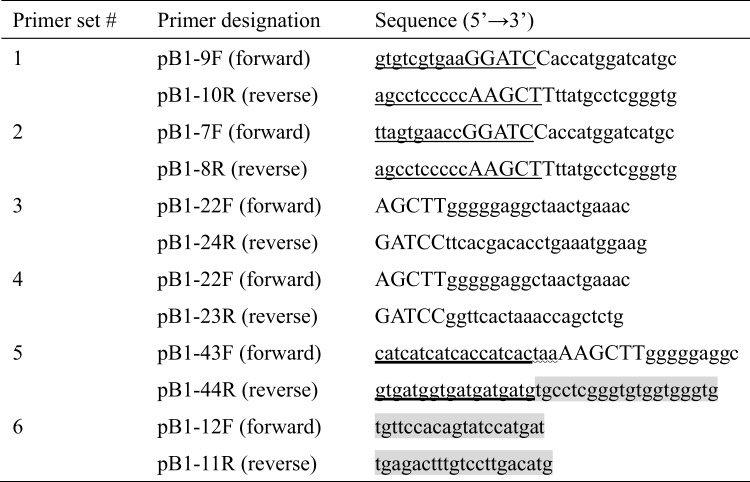
Primer sets used to construct His-tagged prestin expression vectors. Capital letters show the restriction site (or a part of such site). Thin lines show the distinctive 15-base ends for the In-Fusion procedure. Thick lines, the undulating line and the shaded part show 6×His, stop codon and prestin sequences, respectively

### Construction of a stable expression cell line of prestin with His tag

The expression vectors were transfected into CHO cells by lipofection (Lipofectamine 2000, Thermo Fisher Scientific, Waltham, MA) according to the manufacturer’s instructions. The cells were then cultured for approximately 4 weeks in RPMI-1640 medium with 10% fetal bovine serum (FBS) and G418 (G3049, InvivoGen, San Diego, CA) at a concentration of 600 µg/ml at 37 °C with 5% CO_2_ for drug selection. Culture media were changed every 2–3 days during this period. After their selection, the cells were transferred into 96-well plates at a density of 0.8 cells/well using the limiting dilution method. Three plates were prepared and incubated at 37 °C with 5% CO_2_. Single colonies in 96-well plates were picked up and scaled up in RPMI-1640 medium (Sigma-Aldrich) with 10% FBS (Biowest, Nuaillé, France), 100 U penicillin/ml and 100 µg streptomycin/ml (P0781, Sigma-Aldrich) at 37 °C with 5% CO_2_.

### Confirmation and quantification of prestin expression

The expression of His-tagged prestin in the constructed cell lines was confirmed by Western blotting. Briefly, cell proteins were subjected to SDS-PAGE and electroblotted onto a polyvinylidene difluoride (PVDF) membrane (WSE-4051, ATTO, Japan). After blocking with 5% skim milk (190–12865, Wako, Tokyo, Japan) in phosphate-buffered saline (PBS) containing 0.05% Tween 20 (PBS-T), the PVDF membrane was incubated with an anti-6×His-tag mouse monoclonal antibody (66005-1-IG, ProteinTech, Rosemont, IL) diluted at 1:10,000. Bands were visualized using a horseradish peroxidase (HRP)-linked anti-mouse IgG secondary antibody (7076, Cell Signaling Technology, Danvers, MA) diluted at 1:5,000 and a chemiluminescent reagent (Amersham ECL Select Western Blotting Detection Reagent, RPN2235, Cytiva, Marlborough, MA). Images were obtained using a chemiluminescence imager (ChemiDoc, Bio-Rad, Hercules, CA).

The relationship between the chemiluminescent signal intensity of Western blots and the quantity of proteins was examined. As a quantitative reference standard, a 74-kDa multitope protein including a 6×His tag (10 ng/µl, 133–16374, Wako) was used. The protein solution was serially diluted and applied to the gel at approximately 10 µl per well, yielding protein loads of 500, 250, 125, 62.5 and 31.25 pg per lane (i.e., 6.76, 3.38, 1.69, 0.84 and 0.42 fmol per lane, respectively). Based on these samples, a calibration curve was constructed to quantitatively correlate the signal intensity with the protein mass detected by Western blotting. Each sample was then adjusted to a concentration of 100 ng/µl and 15 µl (i.e., 1.5 µg, corresponding to approximately 5.5 × 10³ cells) was loaded into each lane of the gel. To quantify the amount of prestin, 5 µl of the 1600-fold diluted reference standard protein solution (i.e., 31.25 pg) was electrophoresed on the same gel. The region of interest (ROI) surrounding His-tag bands was manually selected as a rectangle and the sum of the intensities of all pixels in the ROI was obtained as the signal for each target. Net intensities were then calculated by subtracting the background intensity of the region without any bands from the sum of the intensities in each ROI. This intensity was then averaged by the number of pixels in each ROI area. Integrated densities were obtained by multiplying the average intensity by the number of pixels in each ROI area. Integrated densities were plotted against concentrations of the reference standard protein. An intensity analysis was performed using Quantity One image analysis software (Bio-Rad) and ImageJ (NIH, Bethesda MD).

### Immunofluorescence experiments

Immunofluorescence experiments were performed to confirm the expression and localization of His-tagged prestin in the constructed cell lines. CHO cells transfected with prestin and untransfected CHO cells were fixed with 4% paraformaldehyde in phosphate buffer at room temperature for 5 min and then washed with PBS. Permeabilization was performed using Triton X-100 (Wako). Cells were then incubated with 2% BlockAce (UKB40, KAC, Kyoto, Japan) at room temperature for 30 min. After washing with PBS, permeabilized samples were incubated with the anti-6×His-tag mouse monoclonal antibody (ProteinTech) diluted 1:10,000 in PBS at 4 °C overnight. They were washed with PBS and incubated with a Cy3-conjugated anti-mouse IgG secondary antibody (A10521, Thermo Fisher Scientific) diluted 1:1,000 in PBS at room temperature for 1 h. Samples were washed with PBS and immunofluorescence images were obtained using a fluorescence microscope (BZ-X800, Keyence, Osaka, Japan).

To evaluate differences in prestin expression among the clones, fluorescence images of all samples were acquired under identical imaging conditions, including the exposure time, gain and other camera settings. Z-stack imaging was not performed; instead, single-plane fluorescence images were used. The images acquired were analyzed using the image analysis software installed on the BZ-X800 microscope (Keyence). Cell regions were extracted based on the following criteria: areas with a cellular area larger than a predefined threshold (100 µm^2^) were defined as individual cells. In cases where adjacent cells overlapped, a watershed algorithm was applied to separate them. The integrated fluorescence intensity of each extracted cell was calculated, and the average intensity per cell was obtained by dividing this value by the total number of pixels in the cell. The mean and standard deviation of the average fluorescence intensity were then evaluated across all extracted cells. In addition, to evaluate the subcellular localization of prestin expression, sectioning images were acquired using the BZ-X800 microscope (Keyence).

### Functional analysis by whole-cell patch clamp technique

Cells expressing functional prestin have been shown to exhibit bell-shaped NLC in response to a change in their membrane potential (Ludwig et al. 2001; Dallos and Fakler 2002). To obtain NLC, electrodes were pulled from a borosilicate glass tube (TW150-4, World Precision Instruments, Sarasota, FL) using a programmable puller (P97, Sutter Instruments, Novato, CA). The electrodes were filled with an internal solution composed of 140 mM KCl, 3.5 mM MgCl_2_, 5 mM EGTA, 5 mM HEPES and 0.1 mM CaCl_2_, with pH adjusted to 7.2 using KOH. Cells were plated onto a 35-mm glass-bottomed dish (IWAKI, Chiba, Japan) with an external solution composed of 145 mM NaCl, 5.8 mM KCl, 1.3 mM CaCl_2_, 0.9 mM MgCl_2_, 10 mM HEPES, 0.7 mM Na_2_HPO_4_ and 5.6 mM glucose, with pH adjusted to 7.2 using NaOH. Measurements of membrane capacitance were performed using a patch amplifier (Axon Axopatch 200B, Molecular Device, San Jose, CA) with the membrane test feature of pCLAMP 8.0 data acquisition software (Molecular Device), as previously described (Iida et al. 2005). To assess the voltage dependence of membrane capacitance, the holding potential was swept from − 140 to 70 mV. After measurements, membrane capacitance was plotted versus membrane potential. Membrane capacitance recorded was fit with the first derivative of the Boltzmann function (Santos-Sacchi 1991),

1 $${C_{\mathrm{m}}}(V)={C_{{\mathrm{lin}}}}+\frac{{{Q_{{\mathrm{max}}}}}}{{a{e^{\frac{{V - {V_{1/2}}}}{\alpha }}}{{\left( {1+{e^{\frac{{V - {V_{1/2}}}}{a}}}} \right)}^2}}}$$

where *C*_lin_ is linear capacitance, *Q*_max_ is maximum charge transfer, *V* is membrane potential, *V*_1/2_ is the voltage at which the maximum charge was equally distributed across the membrane and *α* is the slope factor for the voltage dependence of charge transfer. Charge density, i.e., the expression level of prestin in the unit cell membrane, may also be obtained by dividing *Q*_max_ by *C*_lin_, where *Q*_max_ reflects the expression level of prestin in the whole cell membrane and *C*_lin_ is a linear component of capacitance, which is proportional to the membrane area of the cell.

### Statistical analysis

Immunofluorescence intensities were analyzed using a one-way analysis of variance (ANOVA) followed by Dunnett’s *post hoc* multiple comparison. Data are presented in the text and Figures as the means ± SD. *P*-values < 0.05 were considered to be significant. All statistical analyses were performed with EZR (Saitama Medical Center, Jichi Medical University, Saitama, Japan) (Kanda 2013), which is a graphical user interface for R (The R Foundation for Statistical Computing, Vienna, Austria).

## Results

### Construction of His-tagged prestin expression vectors

Sequence analyses were performed to confirm the prestin expression vectors constructed. The sequences of the N and C termini of prestin obtained from the vectors with EF1α or CMV promoters are shown in Fig. S1 (Supplementary information). The upstream region of prestin and the downstream region containing the 6×His tag were both confirmed to have the expected sequences as designed.

### Construction of His-tagged prestin-expressing CHO cell lines

Cells were transferred from culture dishes to three 96-well plates after transfection. Single colonies of CHO cells transfected with the EF1α expression vector were obtained from 27 wells (9.4%), and were subsequently referred to as EF-1 to EF-27. Single colonies of CHO cells transfected with the CMV expression vector were obtained from 17 wells (6.0%), and were subsequently referred to as CMV-1 to CMV-17.

### Expression of prestin in constructed cell lines

**Fig. 2:**
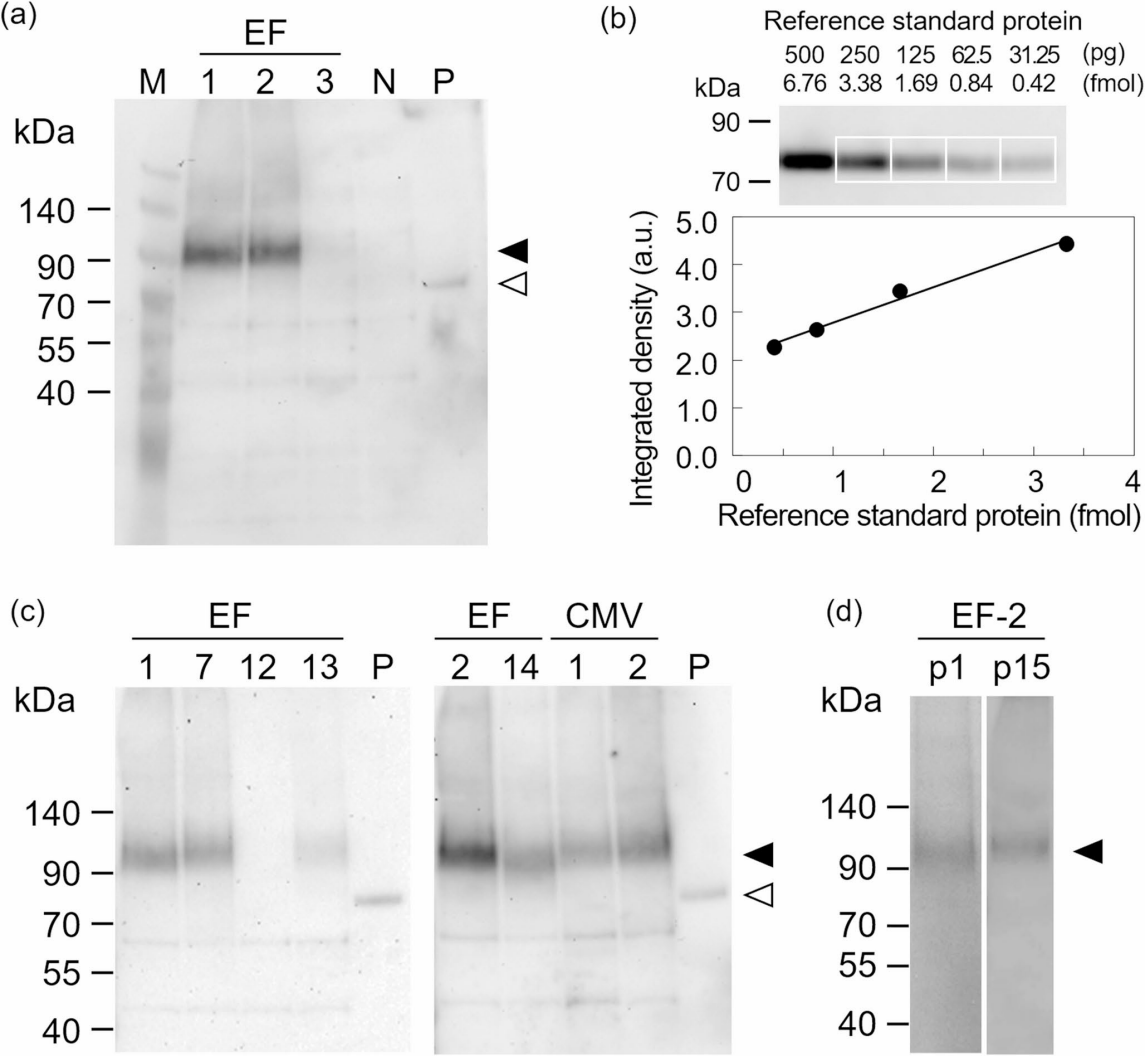
Quantitative Western blot analysis of prestin expression in stable CHO cell lines. (a) Representative Western blot showing His-tagged prestin expression in selected clones. M, marker; EF-1; EF-2; EF-3; N, negative control (untransfected CHO cells); P, positive control (74-kDa reference standard protein including the 6xHis tag; open arrowhead). The expression of prestin (~ 100 kDa) was confirmed in EF-1 and EF-2 (solid arrowhead). (b) Calibration curve generated using known amounts of His-tagged reference protein for absolute quantification. (c) Quantification of prestin expression in constructed CHO cell lines by Western blotting. Analysis was performed of 7 clones expressing His-tagged prestin (EF-1, EF-2, EF-7, EF-13, EF-14, CMV-1 and CMV-2), as well as one clone in which expression was not detected (EF-12) for comparison. P, positive control (reference standard protein including the 6xHis tag; open arrowhead). To quantify the amount of prestin, the samples and reference standard proteins were electrophoresed on the same gel. The expressions of prestin were confirmed at ~ 100 kDa (solid arrowhead). (d) Western blot analysis of prestin expression at early (P1) and late (P15) passages in EF-2, demonstrating stable expression during prolonged culture

An example of the results of the Western blotting analysis is shown in Fig. 2(a). Lanes M, N and P contain the molecular marker, non-transfected CHO cells as a negative control and the multitope protein including the 6×His tag as a positive control, respectively. Three independent clones selected from the 27 clones obtained by transfecting CHO cells with the EF1α expression vector (EF-1, EF-2, EF-3) were loaded to lanes 1 to 3. In lanes 1 and 2 (EF-1 and EF-2), bands were observed at approximately 100 kDa. In contrast, no corresponding band was detected in lane 3 (EF-3). As expected for the negative control, the 100-kDa band was not detected in lane N. In lane P, a band was observed at approximately 74 kDa, which corresponded to the molecular weight of the multitope protein used in this assay. Additionally, weak bands were observed at approximately 40 and 60 kDa in lanes 1 to 3 and lane N.

A Western blotting analysis was performed under the same conditions for all clones. Results are provided in Figs S2, S3 and S4 (Supplementary information). Among the 27 clones generated with the EF1α expression vector and the 17 clones generated with the CMV expression vector, bands corresponding to prestin at approximately 100 kDa were detected in five EF1α clones (EF-1, EF-2, EF-7, EF-13 and EF-14) and two CMV clones (CMV-1 and CMV-2).

Figure 2(b) shows the relationship between the mass of the reference standard protein and the band intensity obtained by Western blotting. Western blot analysis shows bands at approximately 74 kDa, corresponding to the molecular weight of the reference standard protein. White squared areas show the ROI. Since the 500-pg band was saturated, a calibration curve was obtained using the 250-, 125-, 62.5- and 31.25-pg bands. The solid line in the graph indicates the linear regression line *I* = 0.7289*N* + 2.0502 (*r*^2^ = 0.99), where, *I*, *N* and *r* are the integrated density, the amount by mole of the reference standard protein and the correlation coefficient, respectively. The regression coefficient (*r*^2^ = 0.99) indicates a high degree of linearity.

A quantitative analysis was therefore performed on the 7 clones expressing His-tagged prestin, as well as one clone in which expression was not detected (EF-12) for comparison, as shown in Fig. 2(c). The clones of EF-1, EF-7, EF-12 and EF-13 are shown in the left membrane and those of EF-2, EF-14, CMV-1 and CMV-2 in the right membrane. The integrated densities of the 74-kDa band for 31.25 pg of the standard reference protein in lane P were 1.633 and 2.374 in the left and right membranes, respectively. Therefore, the linear regression lines for the left and right membranes were corrected as *I* = 0.7289*N* + 1.3269 and *I* = 0.7289*N* + 2.0679, respectively.

To evaluate the stability of prestin expression, protein levels were compared between early (passage 1) and late (passage 15) passages (Fig. 2(d)). Western blot analysis showed no apparent reduction in prestin expression, indicating stable long-term expression in the established CHO cell line.

Table 2 shows the integrated density values of the 100-kDa bands corresponding to prestin detected in each lane. Using the linear regression equation derived above, the amount by mole of prestin in each lane was quantified and summarized in the table. In the 5 clones constructed with the EF1α expression vector, the average amount of prestin was calculated as 443.0 ± 218.0 pg (*n* = 5). The largest value was 745.0 pg in EF-2. In contrast, in the clones developed with the CMV expression vector, the average amount of prestin obtained was 456.8 ± 100.3 pg (*n* = 2). CMV-2 showed a higher value of 527.7 pg.

**Table 2:** Quantitative analysis of Western blotting on 7 clones expressing His-tagged prestin. In each lane, 15 µl of samples (i.e., approximately 5.5 × 10³ cells) was loaded. The amount by mole of prestin in each clone was calculated using the linear regression equation (Fig. 4) and the corresponding mass was also estimated based on the molecular weight of prestin (81.4 kDa)

	Integrated density (a.u.)	Amount by mole (fmol)	Amount by gram (pg)
EF-1	5.56	5.81	472.6
EF-7	4.92	4.92	400.8
EF-13	2.52	1.64	133.5
EF-2	8.74	9.15	745.0
EF-14	6.22	5.69	463.2
CMV-1	5.52	4.74	385.9
CMV-2	6.79	6.48	527.7

### Immunofluorescence staining of prestin in CHO cells

**Fig. 3:**
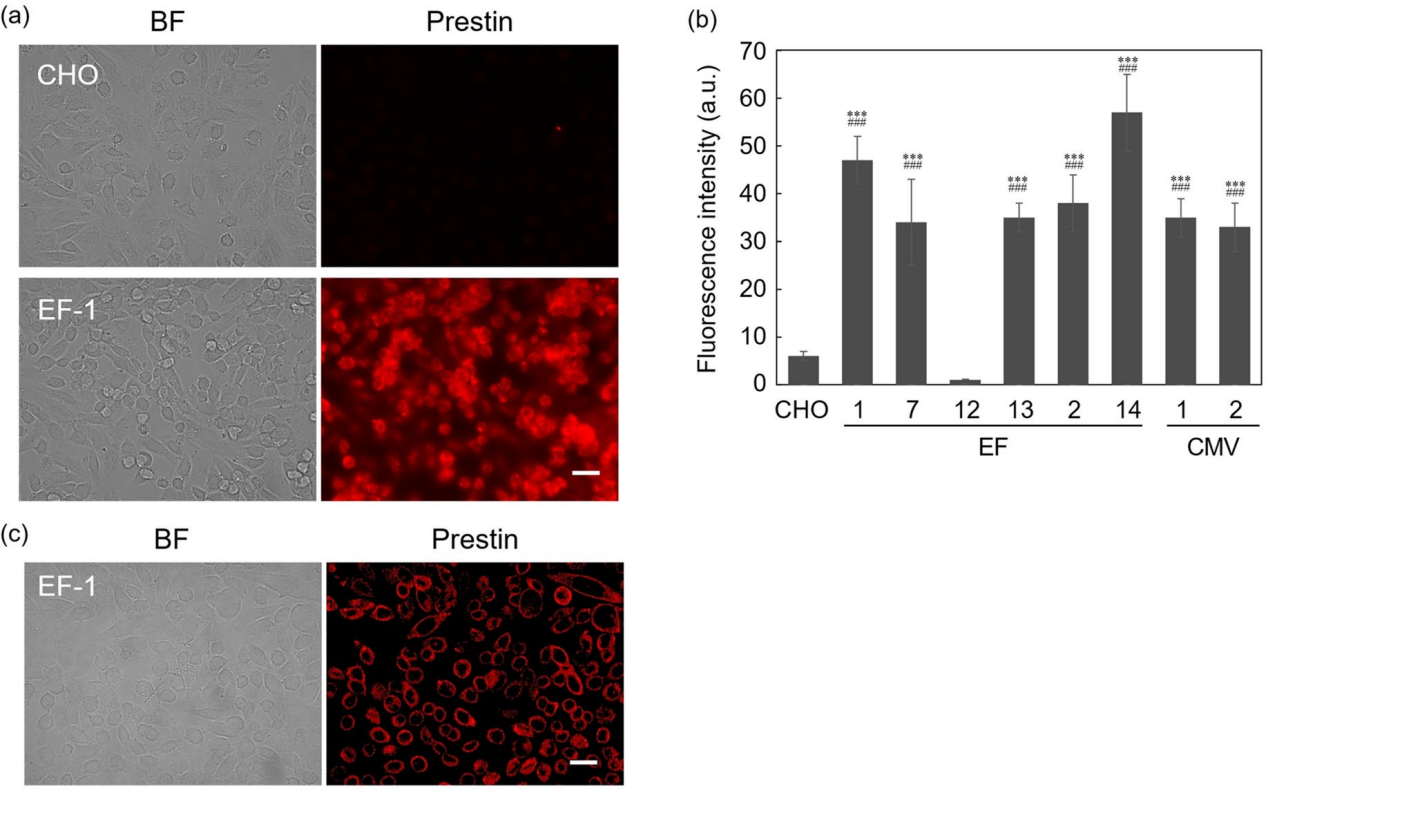
Immunofluorescence analysis of prestin-expressing CHO cell clones. (a) Representative immunofluorescence images showing His-tagged prestin expression in CHO cells. Fluorescence labeling was confirmed in EF-1, but not in CHO cells. BF, bright field image; Prestin, fluorescence image of Cy3 detecting the C-terminal 6×His tag of prestin. The scale bar shows 20 μm. (b) Fluorescence intensities of constructed clones stably expressing His-tagged prestin. The seven clones that exhibited prestin expression in Western blotting showed higher fluorescence intensities than CHO and EF-12. Triple asterisks or triple hashes show statistically significant differences between CHO or EF-12 and each of the seven expressing clones with *p* < 0.001, respectively. (c) A sectioned image of the constructed clone. Fluorescence signal of prestin was predominantly observed near the plasma membrane

The expression of prestin in the established stable cell lines was examined by immunofluorescence staining. As a representative example, the result for EF-1 is shown in Fig. 3(a). While no fluorescence signal was observed in CHO cells, it was evident throughout the cell population of the EF-1 clone, suggesting the expression of prestin in the constructed clone. The results obtained on the other clones are shown in Fig. S5 (Supplementary information).

Immunofluorescence staining was performed to evaluate the expression of prestin in the 7 clones (EF-1, EF-2, EF-7, EF-13, EF-14, CMV-1 and CMV-2) that showed expression by quantitative Western blotting (Fig. 2(c)) and, for comparison, in one clone (EF-12) in which no expression was detected. Based on the fluorescence images acquired, the number of cells was counted using the analysis software of the fluorescence microscope (BZ-X800, Keyence). An example of the result for EF-2 is shown in Fig. S6 (Supplementary information). The number of cells analyzed in each clone was as follows: CHO (*n* = 89), EF-1 (*n* = 99), EF-7 (*n* = 70), EF-12 (*n* = 218), EF-13 (*n* = 121), EF-2 (*n* = 180), EF-14 (*n* = 31), CMV-1 (*n* = 115) and CMV-2 (*n* = 156). The mean and standard deviation of fluorescence intensity for each clone were calculated from the average intensity per cell. As shown in Fig. 3(b), the seven clones that exhibited prestin expression in Western blotting also showed higher fluorescence intensities than CHO and EF-12, which was consistent with the results of Western blotting. Statistical analyses revealed significant differences between CHO or EF-12 and each of the seven expressing clones.

To investigate the subcellular localization of prestin in the established stable cell lines expressing His-tagged prestin, sectioned images of immunofluorescence-stained cells were obtained. As shown in Fig. 3(c), the fluorescence signal corresponding to prestin was predominantly observed near the plasma membrane, suggesting the expression of prestin at the cell membrane in the constructed clones.

### Functional analysis of prestin

**Fig. 4:**
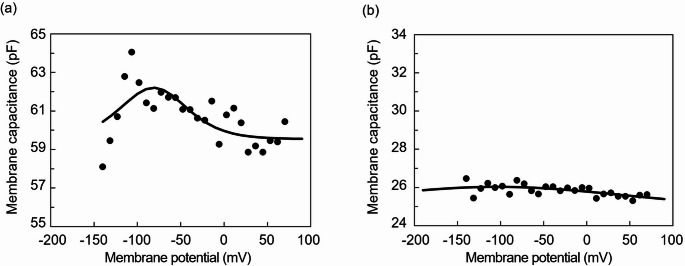
Representative recordings from prestin-expressing and untransfected CHO cells by whole-cell patch clamp. (a) Constructed clone (EF-14). (b) Untransfected CHO cell. Data were fit by Eq. (1) and shown by the solid line. In the constructed EF-14 clone, characteristic non-linear capacitance (NLC) was detected instead of the linear capacitance typically observed in normal cells, suggesting that expressed prestin was functional

A functional analysis of a prestin-expressing clone (EF-14) was performed using whole-cell patch-clamp recordings (Fig. 4). Data were fit according to Eq. (1) and are represented by the solid line. In this cell, instead of the linear capacitance typically observed in normal cells, a characteristic NLC was observed, indicating that the prestin expressed in the constructed clone was functional. The fitting parameters of the derivative of the Boltzmann function were *C*_lin_ = 60.0 ± 0.47 pF, *Q*_max_ = 216.4 ± 59.6 fC, *α* = 25.8 ± 0.04 (mV) and *V*_1/2_ = −81.9 ± 0.65 (mV) and charge density was 225.7 ± 63.8 μm^− 2^ (mean ± standard deviation; *n* = 2). Control recordings obtained from an untransfected CHO cell showed only linear membrane capacitance without detectable nonlinear capacitance.

## Discussion

### Estimation of prestin expression levels in constructed cell lines

The primary goal of this study was to establish a reproducible mammalian expression system for full-length prestin. While prestin expression has previously been achieved using transient mammalian systems, bacterial fragments, insect cells and FLAG- or Avi-tagged constructs, a quantitatively characterized stable CHO expression system compatible with cost-effective affinity purification has remained limited.

We previously established CHO cell lines that stably expressed FLAG-tagged prestin (Iida et al. 2005). In this system, the density of prestin expression estimated from electrophysiological measurements was 255 proteins/µm^2^ per cell. Assuming that a CHO cell is spherical with a radius of 10 μm and considering the molecular weight of prestin, including glycosylation, to be approximately 100 kDa, the total prestin expression level in a 2-L culture containing 2 × 10^9^ cells was estimated to be 106 µg. In the present study, CHO cell lines stably expressing His-tagged prestin were developed. Prestin expression was maintained for at least 15 passages, supporting the stability of the established mammalian expression system (Fig. 2(d)). The prestin expression level was estimated by extrapolating the protein amount detected by SDS-PAGE lanes containing approximately 5.5 × 10³ cells to a large-scale culture equivalent of 2 × 10⁹ cells. Using the EF1α promoter vector, the average expression level was estimated to be 161.1 ± 79.3 µg (*n* = 5; Table 3) with the highest yield of approximately 271 µg observed in the EF-2 clone. In contrast, clones generated using the CMV promoter vector showed an average expression level of 166.1 ± 36.5 µg (*n* = 2), with a maximum of approximately 192 µg observed in CMV-2.

The EF1α promoter produced clones with higher maximum prestin expression than the CMV promoter. This difference may reflect the distinct transcriptional characteristics of these promoters in stable mammalian expression systems. The CMV promoter is known to exhibit strong initial transcriptional activity but may undergo gradual silencing due to DNA methylation and chromatin remodeling after genomic integration. In contrast, the EF1α promoter often provides more stable long-term transcriptional activity across passages and clonal populations. This property may explain why high-producing clones were more readily obtained using the EF1α vector in the present study. The sustained transcriptional activity of EF1α likely contributed to the stable accumulation of prestin in selected clones.

In comparisons with the maximum expression level observed in EF-2 (271 µg), this represents a 2.6-fold increase from that with our previously developed FLAG-tagged prestin expression system. Such an increase in prestin yield compared with the previous FLAG-tagged expression system may result from multiple factors rather than the affinity tag alone. The use of the EF1α promoter likely contributed to improved transcriptional stability after genomic integration. In addition, clonal screening procedures may have enabled the isolation of higher-producing cells. The smaller size of the His tag may also reduce potential interference with protein folding, trafficking or degradation compared with larger peptide tags, although this possibility was not directly tested in the present study.

As for lower-molecular-weight bands observed at approximately 40 and 60 kDa, they may possibly represent truncated or partially degraded forms of prestin, which could arise during expression or sample preparation. Reducing such products may further improve the yield of full-length prestin in future studies.

Taken together, these results suggest that the EF1α promoter is advantageous for establishing high-producing prestin-expressing CHO clones in stable mammalian expression systems.

**Table 3:** The amount by mole of prestin in large-scale samples (i.e., approximately 2.0 × 10^9^ cells) and the corresponding mass were estimated based on this experiment (Table 2)

	Estimated amount of the substance (nmol)	Estimated amount (µg)
EF-1	2.11	171.86
EF-7	1.79	145.75
EF-13	0.60	48.53
EF-2	3.33	270.91
EF-14	2.07	168.45
CMV-1	1.72	140.31
CMV-2	2.36	191.88

### Comparative evaluation of the stable, full-length and mammalian expression system for prestin

In the present study, we established CHO cell lines stably expressing full-length prestin with a C-terminal His tag using EF1α or CMV expression vectors. Sequence verification confirmed correct junctions at the N and C termini, including the 6×His cassette designed for purification and detection. Among the 44 screened clones, Western blotting detected bands at approximately 100 kDa in seven clones, enabling absolute prestin quantification using a validated calibration curve. Immunofluorescence microscopy showed membrane-localized signals throughout the seven clones, whereas the clones that did not express prestin exhibited low background signals. Whole-cell patch clamp recordings revealed NLC in EF-14, demonstrating that the constructed system produced His-tagged, full-length prestin along with its characteristic motor function.

Although previous bacterial systems expressed prestin with the His tag, it was fragments rather than the intact transporter, constraining conclusions about folding and targeting as well as electromechanical competence (Pasqualetto et al. 2008). A subsequent analysis examined the cytosolic portion and showed its crystal structure; however, it still omitted the multi-pass membrane core required for prestin function (Pasqualetto et al. 2010). Bacterial hosts lack a mammalian lipid composition and endomembrane quality control, which guide correct topology and oligomeric assembly in vivo. These differences between bacterial and mammalian systems limit inference about trafficking checkpoints and membrane insertion, possibly affecting coupling between structure and electromechanical output in mammalian cells.

Our system expressed full-length prestin with a His tag in CHO cells, preserving a native membrane context for assembly and targeting. This design for protein expression enabled a direct link among expression, localization and function within a single experimental framework. In addition, the His tag supported immobilized metal affinity chromatography and broad anti-His detection, facilitating purification and assay standardization.

To address these issues, we previously employed an Sf9 insect-cell baculovirus expression system, which enabled rapid production of functional prestin (Tadenuma et al. 2008). Mio et al. (2008) also analyzed negatively stained FLAG-tagged prestin expressed in an insect cell system by cryo-EM and reported a tetrameric, bullet-shaped structure with inner cavities and a prominent cytoplasmic domain (Mio et al. 2008). Although insect cell systems, such as Sf9, offer advantages including rapid expression and relatively high protein yields, their ability to reproduce mammalian-specific post-translational processing and membrane environments is limited. These differences may affect protein stability, structural integrity and functional properties when compared with mammalian expression systems.

A mammalian cell system more accurately models post-translational maturation and subcellular targeting, which are essential for prestin activity, than an insect system, allowing quantitative Western blotting, immunofluorescence imaging and electrophysiological measurements on an identical full-length construct. Previous mammalian studies used transient transfection to examine the assembly of prestin under various expression levels (Zheng et al. 2006). Transient expression introduced batch effects and may shift apparent trafficking efficiency and distort comparisons of membrane delivery between experiments and clones.

Genomically integrated clones reduced this variability and supported stable expression, enabling standardized inputs for downstream experiments. Stable expression facilitated longitudinal comparisons across plates and passages without the repeated optimization of transfection conditions. These attributes are important for the rigorous quantification of expression and NLC within one system. We previously established CHO lines stably expressing prestin with a FLAG tag and demonstrated feasibility for its purification and supported its downstream biochemical characterization (Iida et al. 2005, 2008).

In the present study, the system adopting a C-terminal His tag was developed to leverage widely available resins and anti-His tools for detection and purification. His tag-based workflows simplify technology transfer, reduce specialized reagent needs and enable consistent protocols across laboratories and instruments. We also implemented EF1α and CMV promoters to increase the likelihood of isolating high-producer clones with uniform behavior. Promoter diversification enabled efficient screening and selection while preserving the full-length coding sequence and native topogenic signals.

Modern structural biology, such as cryo-EM, and related analyses increasingly depend on reliable expression systems capable of producing homogeneous membrane-protein samples. Thermostabilized constructs and structural analyses are most effective when proteins are produced in cellular environments that preserve protein integrity and native-like conformational states (Bavi et al. 2021; Ge et al. 2021; Butan et al. 2022; Futamata et al. 2022). The present system provides a practical basis for such studies by enabling stable expression of full-length prestin in mammalian cells while maintaining appropriate membrane composition and trafficking processes. This compatibility allows integration with structural biology workflows while preserving functional properties of prestin for parallel biochemical, imaging and electrophysiological assays on the same construct.

From a cell engineering perspective, the present study demonstrates that stable mammalian expression combined with quantitative screening is an effective strategy for producing functional multi-pass membrane proteins.

### Limitations of present study

This study has several limitations that should be considered when interpreting the results. First, CHO cells differ from native outer hair cells in their lipid environment and associated membrane proteins, which may influence prestin behavior to some extent. Second, although functional prestin activity was confirmed by nonlinear capacitance (NLC) measurements in the EF-14 clone, the number of electrophysiological recordings was limited. Future studies should include additional measurements across multiple high-expressing clones to further validate the functional stability of prestin in this expression system.

Despite these limitations, the present system offers practical advantages for prestin research. This mammalian expression system, which is full-length, His-tagged and stably integrated, addresses limitations associated with bacterial fragments, insect hosts and transient expression systems. Furthermore, it extends earlier FLAG-tagged CHO expression studies by broadening purification options and facilitating the identification of high-producing clones through promoter diversification (Iida et al. 2005, 2008). The system enables reproducible expression and quantitative evaluation within a single cellular background and will support future studies on the relationship between the structure and function of prestin.

## Conclusions

The present study highlights the utility of CHO cells as a host for the stable production of functional membrane proteins. We established CHO cell lines stably expressing His-tagged prestin. Among the clones generated, the EF1α promoter–driven system achieved the highest protein yield, approximately 271 µg per 2 × 10⁹ cells, representing a 2.6-fold increase compared with our previously developed FLAG-tagged prestin expression system in CHO cells. The expression and membrane localization of prestin were confirmed by Western blotting and immunofluorescence analyses, and functional activity was demonstrated electrophysiologically by measuring NLC. These results indicate that CHO cells provide a robust and versatile system for the stable production of His-tagged prestin, enabling efficient purification using cost-effective methods. This system is expected to facilitate future structural and functional studies of prestin and contribute to a better understanding of the molecular mechanisms underlying cochlear amplification.

## Supplementary Information

Below is the link to the electronic supplementary material.

Supplementary Material 1

## Data Availability

All datasets are available in the figshare repository at https://doi.org/10.6084/m9.figshare.30013903, https://doi.org/10.6084/m9.figshare.30014410 and https://doi.org/10.6084/m9.figshare.30014431.
