# Supplementary material for: Stable mammalian expression of His-tagged prestin in Chinese hamster ovary cells
Source: Cytotechnology. 2026 Apr 10;78(3):83. doi: 10.1007/s10616-026-00950-8 (PMC13069067; doi:10.1007/s10616-026-00950-8)
Supplement: Supplementary file 1 — Supplementary Material 1 [file 10616_2026_950_MOESM1_ESM.pdf]

Title: Stable mammalian expression of His-tagged prestin in Chinese hamster ovary cells
Journal: Cytotechnology
Authors: Yasunori Donjo[1,2], Hisashi Sugimoto[1], Ryosei Motoo[1,2], Manabu Inaba[1,2], Tomokazu Yoshizaki[1], Michio Murakoshi[2*]
[1] Department of Otolaryngology-Head and Neck Surgery, Kanazawa University, Kanazawa, Japan.
[2] Faculty of Frontier Engineering, Institute of Science and Engineering, Kanazawa University, Kanazawa, Japan.
----
*Corresponding Author:
Michio Murakoshi, Ph.D.
E-mail: murakoshi@se.kanazawa-u.ac.jp

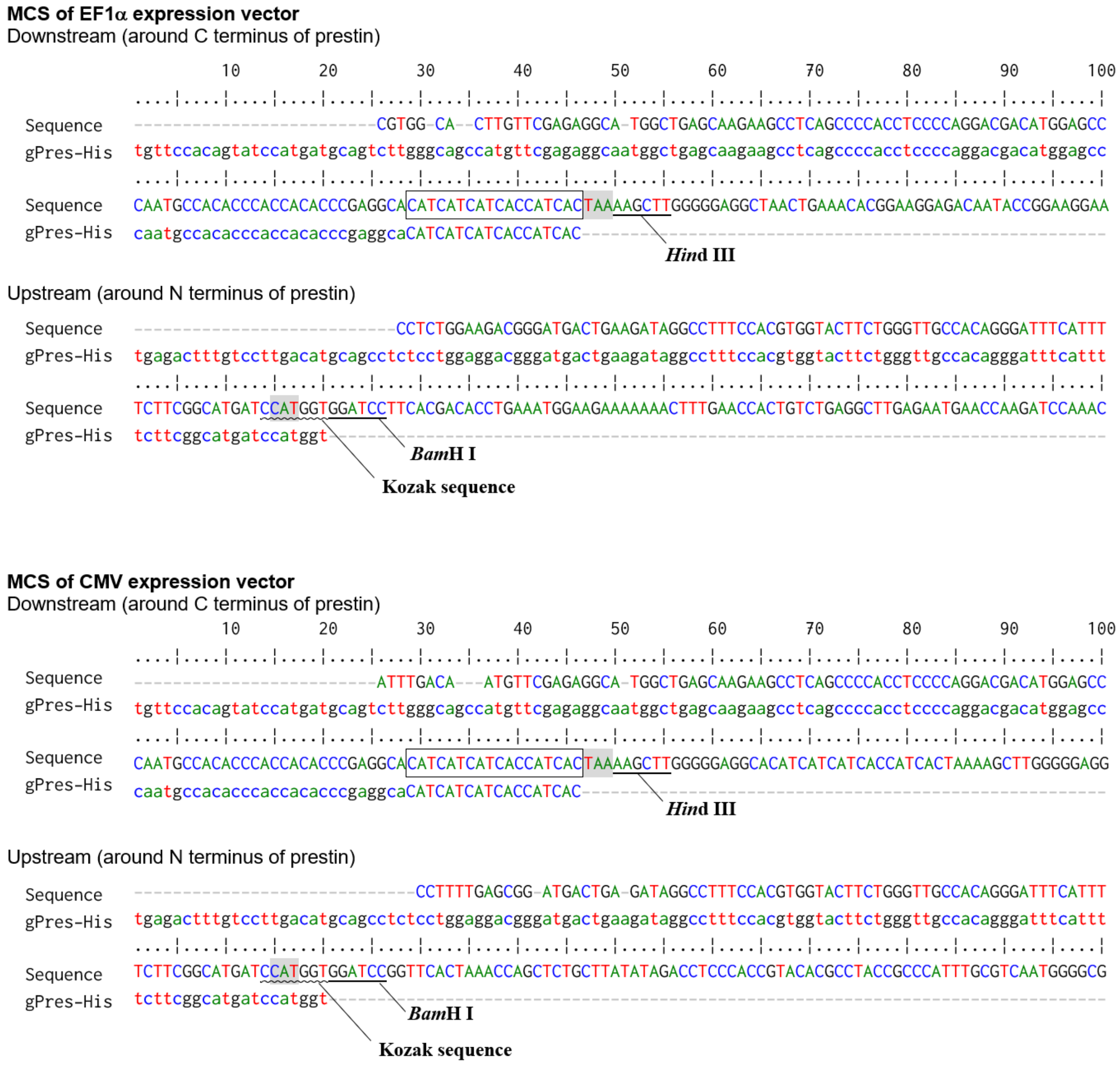

Fig. S1 The Sequences around C and N termini of prestin obtained from constructed expression vectors with EF1α or CMV promoters. In the downstream region (around the C terminus of prestin), boxed regions show the 6xHis tag followed by stop codons (TAA). The underlined regions show the *Hind* III restriction site of the MCS of the expression vector. In the upstream region (around the N terminus of prestin), the wavy underlined region shows the Kozak sequence of prestin and the underlined region shows the *BamH* I restriction site of the MCS.

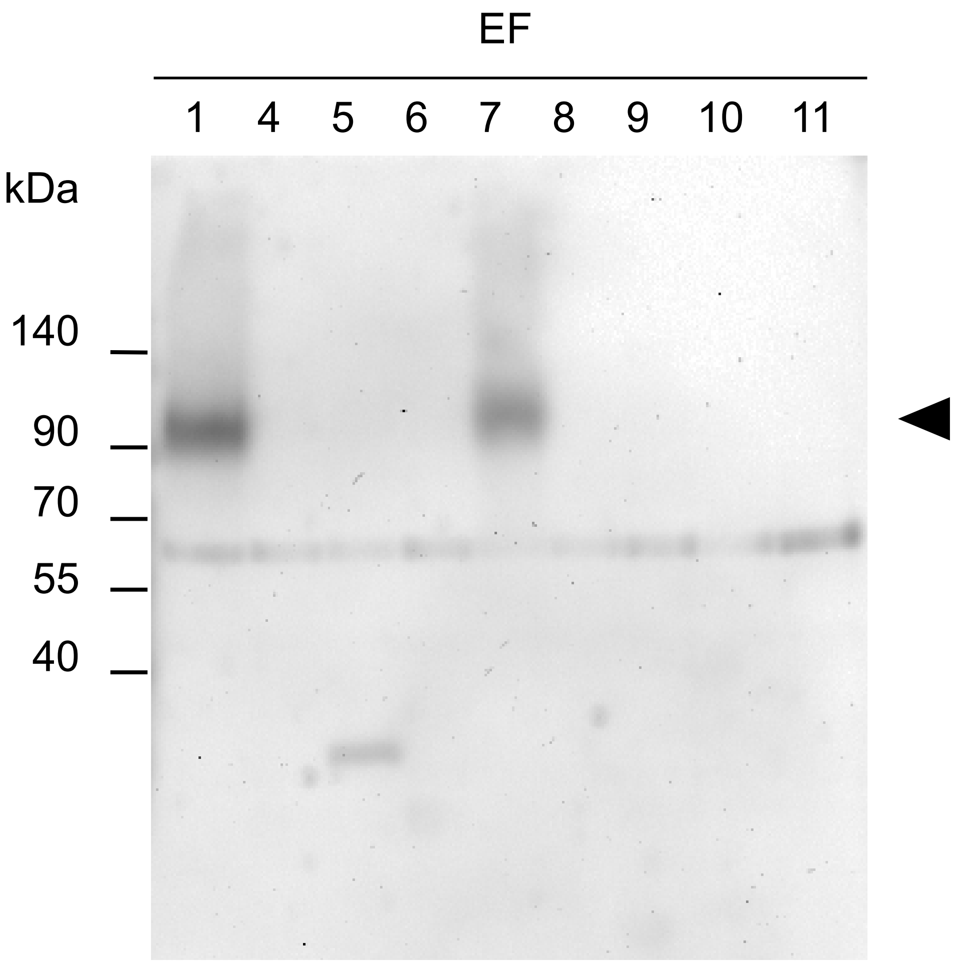

Fig. S2 Western blot analysis of prestin expression in constructed CHO cell lines transfected with the EF1α expression vector. In all clones, Western blotting was performed under the same conditions. The expression of prestin was confirmed in EF-1 and EF-7 at approximately 100 kDa (solid arrowhead).

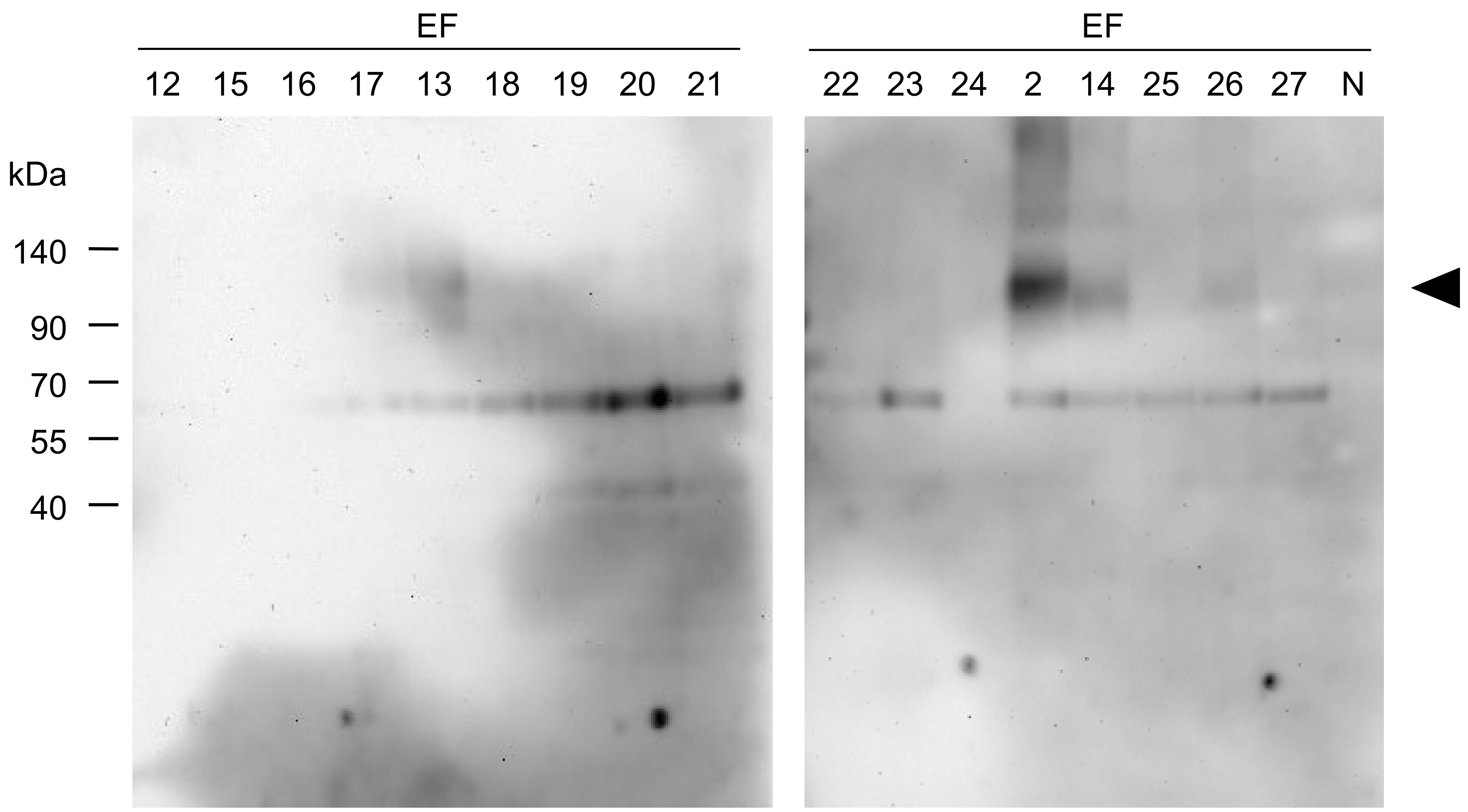

Fig. S3 Western blot analysis of prestin expression in constructed CHO cell lines transfected with the EF1α expression vector. In all clones, Western blotting was performed under the same conditions. The expression of prestin was confirmed in EF-13, EF-2 and EF-14 at approximately 100 kDa (solid arrowhead). N, negative control (untransfected CHO cells).

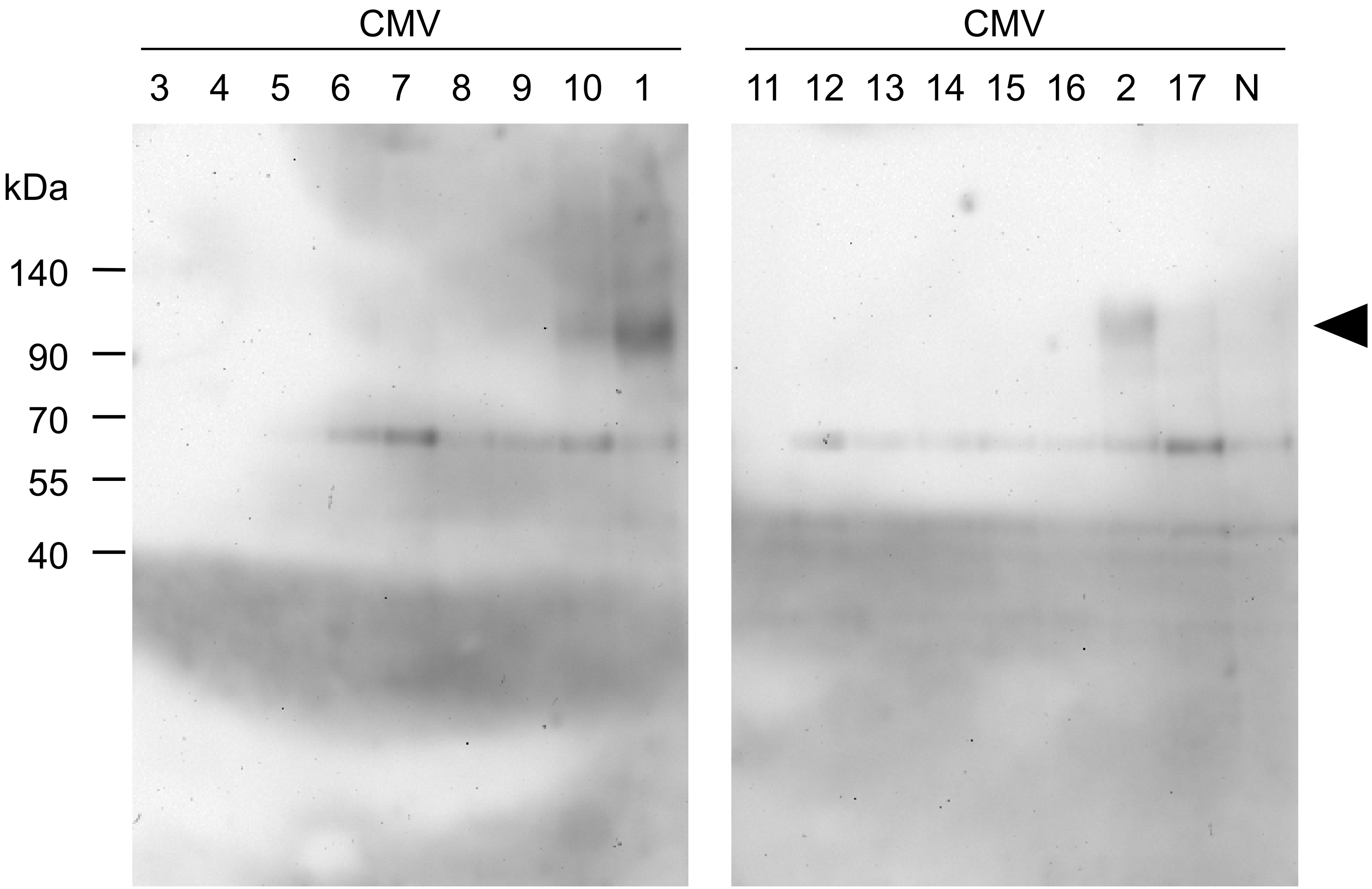

Fig. S4 Western blot analysis of prestin expression in constructed CHO cell lines transfected with the CMV expression vector. In all clones, Western blotting was performed under the same conditions. The expression of prestin was confirmed in CMV-1 and CMV-2 at approximately 100 kDa (solid arrowhead). N, negative control (untransfected CHO cells).

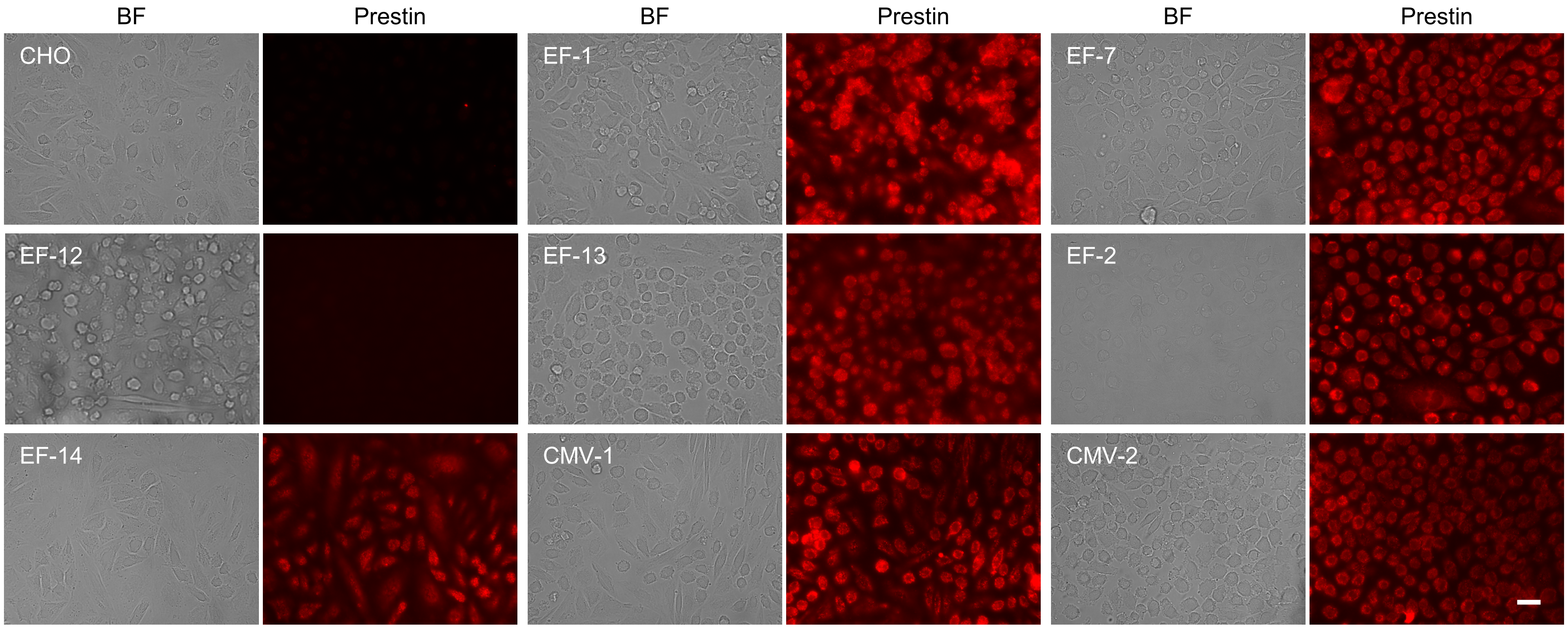

Fig. S5 Expression of prestin in constructed clones of CHO cells detected by an immunofluorescence analysis. Fluorescence labeling was confirmed in the 7 clones (EF-1, EF-2, EF-7, EF-13, EF-14, CMV-1, and CMV-2), but not in CHO cells or in one clone (EF-12). BF, bright field image; Prestin, fluorescence image of Cy3 detecting the C-terminal 6 × His tag of prestin. The scale bar shows 20 μm.

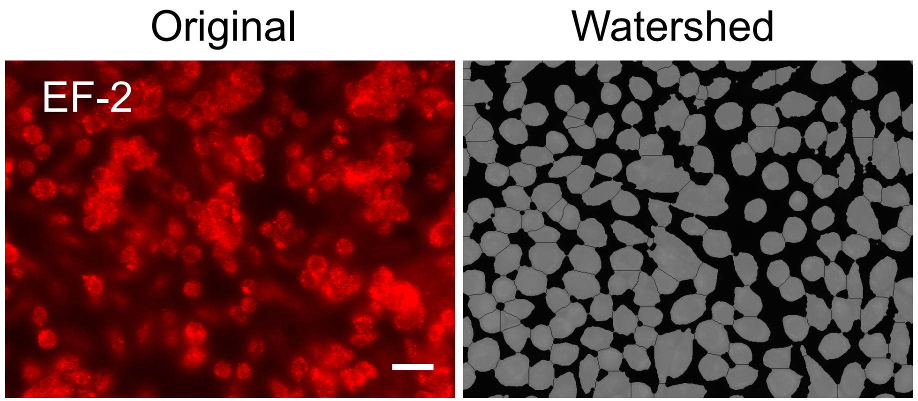

Fig. S6 Representative results of cell counting using BZ-X800 analysis software. Based on the fluorescence image, the number of cells was counted. In cases where adjacent cells overlapped, a watershed algorithm was applied to separate them. The scale bar shows 20 μm.
